# Challenges and Advances in the Encapsulation of Bioactive Ingredients Using Whey Proteins

**DOI:** 10.3390/foods14040691

**Published:** 2025-02-17

**Authors:** Manuel Figueiredo, Zsuzsa Sárkány, Fernando Rocha, Pedro M. Martins

**Affiliations:** 1 LEPABE—Laboratory for Process Engineering, Environment, Biotechnology and Energy, Faculty of Engineering, University of Porto, Rua Dr. Roberto Frias, 4200-465 Porto, Portugal; 2 ALiCE—Associate Laboratory in Chemical Engineering, Faculty of Engineering, University of Porto, Rua Dr. Roberto Frias, 4200-465 Porto, Portugal; 3 i3S—Instituto de Investigação e Inovação em Saúde, Universidade do Porto, 4150-180 Porto, Portugal; 4 IBMC—Instituto de Biologia Molecular e Celular, Universidade do Porto, 4200-135 Porto, Portugal

**Keywords:** whey protein, β-lactoglobulin, binding, encapsulation, nanocarriers, bioactive compounds, nutraceuticals

## Abstract

Functional foods represent an emerging trend in the food industry. Fortifying foods with bioactive ingredients results in health benefits and reduces the risk of disease. Encapsulation techniques protect sensitive ingredients from degradation due to heat, light, moisture and other factors. Among encapsulating materials, milk whey proteins are particularly attractive due to their availability, GRAS status and remarkable ligand-binding ability. Whey protein was once considered a by-product in the dairy industry but is now seen as a promising resource given its natural role as a nutrient carrier. This work reviews the encapsulation systems that employ whey proteins in the food industry. The structural features of β-lactoglobulin (β-LG), the main protein constituent of milk whey, are presented in the context of its ligand-binding properties. Different types of encapsulation systems using whey proteins are discussed, focusing on the recent advances in stable formulations of bioactives using whey protein, alone or in hybrid systems. Whey proteins are a valuable asset capable of binding sensitive bioactive compounds such as vitamins, polyphenols and antioxidants and forming stable complexes that can be formulated as nanoparticles, nanofibrils, emulsions and other micro- and nanostructures. Developing scalable, solid and stable encapsulation systems is identified as a main challenge in the field.

## 1. Introduction

Consumers are increasingly conscious about the importance of a healthy diet, which has motivated the emerging trend of functional foods. There is a growing interest in fortifying foods with bioactive ingredients such as vitamins and polyphenols. Many such ingredients are limited in their use as supplements due to their poor solubility in water, fast degradation, sensitivity to light, heat, moisture, oxygen, and other factors [1,2]. Bioactive compounds must be kept stable during food processing, storage and in the gastrointestinal tract until they are absorbed, to ensure their effectiveness. To protect sensitive ingredients from degradation, both during processing and during storage and to improve organoleptic properties, encapsulation strategies are employed [3,4].

Encapsulation consists of the entrapment or embedding of one or more active ingredients in a continuous matrix of the encapsulating material (encapsulating agent) [3,4]. Often, encapsulation also has the added advantage of converting a liquid feed into a more easily handled solid powder. Different chemical, physical and physicochemical encapsulation techniques are described in the literature, envisaging the preparation of a great variety of micro- and nanostructures. Many of the widely used encapsulation techniques, such as spray drying, involve high temperatures and other harsh conditions that can cause the degradation of the bioactive compound [5]. This is a driver for research into novel, milder encapsulation techniques.

The choice of encapsulating agent has a major impact on the properties of the product. For application in the food industry, the choice of materials for encapsulants is constrained by concerns about safety and secondary effects, as well as the most recent trends in consumer preferences [6,7]. This has motivated research into novel encapsulation strategies using natural biopolymers (carbohydrates, lipids and proteins) and hybrid systems combining different biopolymers [8]. Polysaccharides can suffer from poor stability against pH and temperature changes. Lipids are prone to oxidation and other types of degradation. Synthetic polymers raise concerns about their biocompatibility, toxicity and degradation pathways [9]. Food proteins are very promising choices for encapsulating materials, owing to their safety, their natural ability to bind ligands and their capacity to self-assemble into different nanostructures [10].

Whey is the liquid part of milk that is obtained from the precipitation and removal of solid curds during cheese production. The main components of whey are water, lactose, proteins and minerals [11]. In the past, whey has been regarded as a by-product of the dairy industry, requiring expensive treatments before it could be discarded as waste. Whey proteins have found interest as an ingredient in the food industry not only for their nutritional value, but also for their useful functional properties as an emulsifier, texture modifier and their remarkable ligand-binding ability [12]. Whey proteins are relatively inexpensive, can be easily concentrated and purified, and are “Generally Regarded As Safe” (GRAS) [7,13].

The main protein constituent of milk whey is β-lactoglobulin (β-LG). β-LG has multiple binding sites within its structure and has been shown to bind a wide variety of known bioactive compounds through non-covalent interactions [14,15]. Binding to β-LG has been shown to slow the decomposition or loss of activity of several bioactive compounds, such as vitamins [16,17] and phenolics [18]. The other milk proteins α-lactalbumin (α-La) and bovine serum albumin (BSA) also have ligand binding sites [19]. These interactions between whey proteins and bioactive ingredients have been the object of recent studies aimed at developing them into stable delivery systems, both in the form of molecular complexes and in the form of supramolecular assemblies, such as nanocarriers [15,20]. While using pure or nearly pure β-LG may be too expensive for application in the food industry, commercial products including whey protein concentrate (WPC) and whey protein isolate (WPI) are available, which contain respectively 50–85% and >90% protein [21].

This article is a review of the encapsulation systems based on whey proteins, specifically focusing on β-LG. The first sections outline the structure and properties of β-LG and its ligand-binding mechanisms and how they justify its potential as an encapsulating agent. Next, the different types of molecular and supramolecular whey protein encapsulation systems are discussed. Recent achievements in whey protein + ligand formulations are reviewed and discussed, with an emphasis on stability and a view towards practical applications.

## 2. β-Lactoglobulin Structure

The most abundant protein in cow milk whey is β-LG, which makes up 50% of whey protein mass [21]. β-LG is found in the milk whey of several animals, although it is notably absent from human milk [22]. The precise biological function of β-LG remains controversial, but it has been reported to bind a variety of compounds, mainly small hydrophobic molecules, such as vitamins, polyphenols and fatty acids [11,23].

Structurally, β-LG is a very well characterized protein. It is a small globular protein, which exists predominantly as a homodimer and tends to dissociate at low pH [22,24]. In the case of bovine β-LG, each subunit consists of 162 amino acid residues and has a molecular weight of 18.3 kDa. Bovine β-LG has as many as nine genetic variants, with A and B being the most common and widely studied. Variants A and B are only distinct in two amino acid residues: Asp-64 and Val-118 in variant A are replaced by Gly-64 and Ala-118 [24,25]. It has an isoelectric point of Section 5.3.

**Figure 1:**
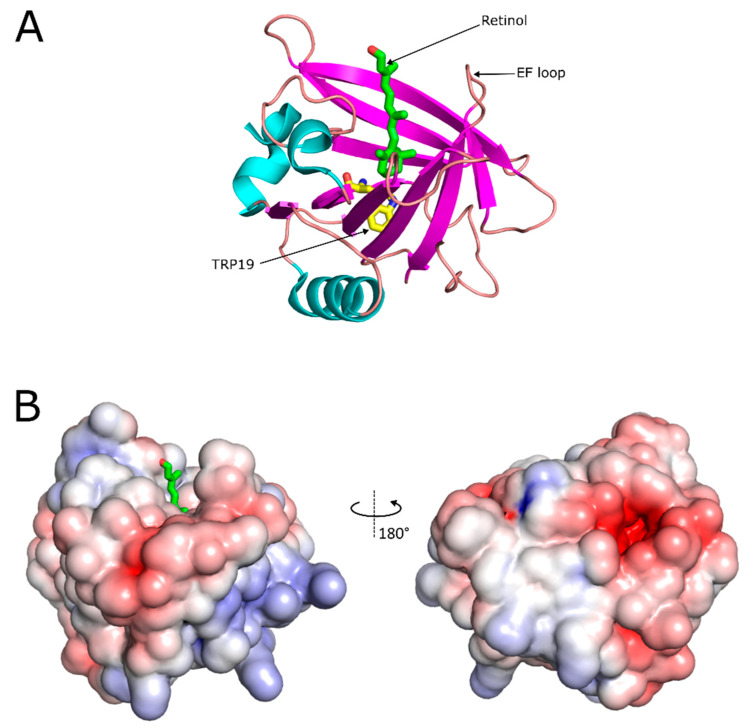
Retinol binds into the calyx, the main hydrophobic binding site of β-LG. (**A**) The overall crystal structure of monomeric β-LG variant B in complex with retinol (PDB entry 1GX8). The β-LG structure is represented as cartoon where the colors highlight different types of secondary structures. The main fluorophore TRP19 is indicated at the bottom of the calyx as yellow sticks. Retinol is denoted as green sticks. (**B**) Electrostatic surface representation of β-LG. The surfaces are colored according to electrostatic potential contoured at ±8 kT/eV (blue, positive; red, negative). Left and right panels are related by 180° rotation. Images of β-LG: retinol complexes were prepared with Pymol (The PyMOL Molecular Graphics System, Version 2.5.0 Schrödinger, LLC, New York, NY, USA).

The structure of each β-LG monomer (shown in Figure 1) consists of one α-helix (8%), nine strands of β-sheet (~45%) and random coil (~47%) [25]. Eight of the β-strands (A-H) are arranged in two antiparallel sheets, which form a β-barrel. The ninth β-strand is a part of the dimer interface [14,22]. The β-barrel is a cylindrical hydrophobic cavity, often referred to as a calyx, with a length of 1.5 nm and is the primary binding site for many bioactives, in particular for small hydrophobic molecules, such as most vitamins. Because of this characteristic fold, β-LG belongs to the family of lipocalins [14]. β-LG has two disulfide bridges (Cys-66/Cys-160 and Cys-106/Cys-119) and an additional free thiol at Cys-121 [14,26].

The conformation of β-LG undergoes several changes with the pH. Close to physiological pH, between 6.5 and 8.0, β-lactoglobulin undergoes a conformational transition, known as the Tanford transition, which involves the rearrangement of the EF-loop. The EF-loop forms a flap located at the “mouth” of the calyx, acting as a gate to the binding site. When the pH is below the transition threshold, the backbone of the EF-loop lies parallel to the opening of the hydrophobic cavity and covers it. In this “closed” configuration, the EF-loop forms hydrogen bonds. The protonation of the glutamate residue Glu-89 causes the cleavage of the hydrogen bonds and triggers a conformational change, where the EF-loop flap flips “open”. In the “open” configuration, the hydrophobic interior of the β-barrel is accessible as a binding site [26,27,28]. It has been hypothesized that this “closed“ conformation protects the ligand from the acid environment in the stomach, to be later released in the small intestine, where the protein adopts the “open” conformation [27,29]. This, in addition to β-LG’s good aqueous solubility, high resistance to low pH and proteolysis in the stomach, suggests its application to protect hydrophobic molecules during passage through the stomach [6,11].

## 3. β-Lactoglobulin Ligand-Binding Properties

The native structure of β-LG possesses several patches which act as binding sites, leading to the formation of protein–ligand molecular complexes. Binding is mostly driven by hydrophobic interaction between ligands and hydrophobic patches on the protein surface, although other intermolecular forces, such as hydrogen bonds, are also involved [7,30]. Such molecular complexes can be prepared under mild conditions by the simple process of mixing solutions of the protein and ligand in adequate solvents [20]. Hydrophobic ligands, such as retinol and resveratrol, are dissolved in non-aqueous solvents. Care must be taken in the preparation of the complexes, so that the final concentration of non-aqueous solvent is low and does not affect the protein structure.

**Figure 2:**
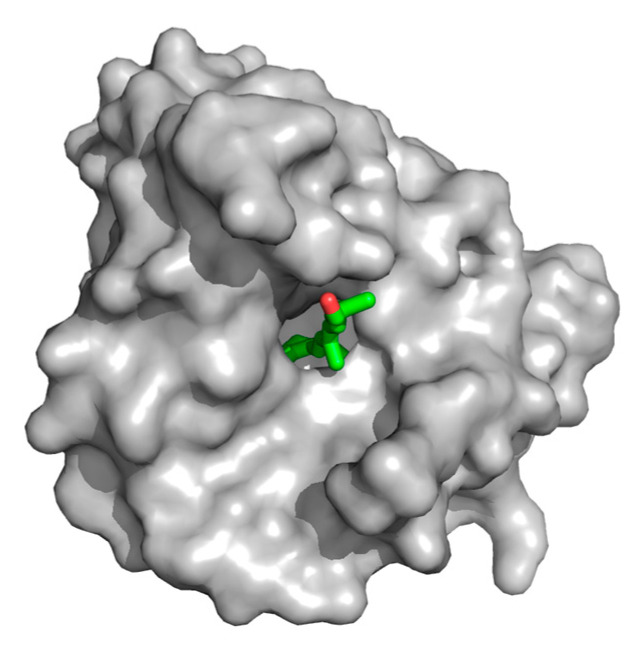
Surface representation of monomeric β-LG variant B in complex with retinol (PDB entry 1GX8). Retinol is denoted as green sticks. Images of β-LG: retinol complexes were prepared with Pymol (The PyMOL Molecular Graphics System, Version 2.5.0 Schrödinger, LLC).

β-LG has been reported to bind more than 200 different compounds [25]. The binding location, stoichiometry and affinity depend on the chemical nature of each individual ligand. The main binding site for hydrophobic ligands is the inside of the central calyx, which is lined with hydrophobic residues, except for two lysine residues [14,31]. Figure 2 shows the main binding site and how ligands fit into the inside of the calyx.

Researchers have described varying numbers and locations of secondary binding sites. In addition to the inside of the β-barrel, three binding sites have been most consistently reported [32,33]:

- At the surface hydrophobic pocket in the groove between the α-helix and β-barrel.
- At the outer surface near Trp-19/Arg-124.
- At the dimer interface.

Other binding sites have been proposed, such as the outer surface of the β-barrel and close to the opening of the calyx [33]. Site-directed mutagenesis has also been used to create β-LG variants capable of binding other bioactive compounds such as the anti-depressant desipramine [33] and the anesthetic pramoxine [34].

At pH below 2.7, β-LG dissociates into monomers and the binding site at the dimer interface disappears. On the other hand, dissociation into monomers causes more regions on the protein surface to become available [33].

Retinol is the most extensively studied β-LG ligand, known to bind to the internal site with a 1:1 stoichiometry, and it is used as a reference ligand in studies of other ligands [35,36]. Competition of other ligands with retinol is taken as evidence of their binding to the central calyx, as is the case with α-tocopherol (vitamin E) [37]. Palmitate is also known to bind the central calyx and is also used in competitive binding experiments [38]. Crystal structures of β-LG complexes with fatty acids with different hydrocarbon chain lengths showed that fatty acids bind the central calyx, with the exact position varying with the chain length [23,39].

On the other hand, molecules such as carotenoids [40] and folic acid [41] do not seem to compete for the calyx site, indicating affinity for an alternate binding site. Some hydrophobic compounds preferentially bind the central calyx with a 1:1 stoichiometry, but they have been shown to bind secondary binding sites when in molar excess or when the primary binding site is inaccessible [42,43,44]. The availability of binding sites can be affected by changes to the protein structure, which can be brought about by factors such as temperature, pH, ionic strength, as well as the binding of other ligands [45]. For example, at acid pH, the closed conformation of the EF-loop makes the main binding site less accessible to ligands. X-ray crystallography structures of β-LG complexes with sodium dodecyl sulfate (SDS) at pH 4.5 showed SDS bound inside the β-barrel, but also an additional SDS molecule bound near the dimer interface, albeit with low affinity [27].

The formation of a stable complex at acidic pH has been taken as an indication that some compounds, such as phenolics, can bind to sites other than the central calyx [35]. In a 2012 study, Liang and Subirade showed that tryptophan fluorescence quenching induced by the binding of resveratrol was similar at neutral and acid pH. Since binding at the internal cavity is disrupted due to the acid pH, the authors concluded that the ligand must use a different binding site which remained stable [32].

Heat treatment causes the protein to unfold, which can be measured by fluorescence spectroscopy. After prolonged heat exposure, the fluorescence emission peak of β-LG will approach that of free tryptophan, indicating its unfolded state [46,47]. When the calyx unfolds, the protein becomes unable to accommodate ligands inside it. Yang et al., found that vitamin D retained 35% of its quenching effect on β-LG fluorescence at low pH and 40% of its quenching effect on heat-treated β-LG, when compared to native β-LG. This indicates a second binding site more resistant to heat and pH. The authors determined that the secondary binding site for vitamin D is near the COOH-terminus, in a cleft next to the α-helix and the β-strand I, which is involved in the dimer interface [43]. Recently, Ghosh et al., used the novel method of protein NMR spectroscopy to study the perturbations caused by the binding of curcumin and folic acid at different pHs. At eight times molar excess and pH 6.5, curcumin and folic acid were found to bind both the central calyx and the secondary site at the groove between the α-helix and β-barrel. At pH 2, curcumin was bound only to the secondary site, while the exact binding site for folic acid could not be ascertained [48].

## 4. Methods to Investigate Ligand Binding

### 4.1. Intrinsic Fluorescence Spectroscopy

Fluorescence spectroscopy has been extensively used to study protein–ligand interactions. Protein intrinsic fluorescence arises from the three aromatic amino acid residues, tryptophan (Trp), tyrosine (Tyr) and phenylalanine (Phe), where Trp dominates over the other two, particularly at excitation wavelengths above 290 nm. β-LG has intrinsic fluorescence due to four Tyr and two Trp residues. The Trp-61 residue is responsible only for a minor contribution to the overall fluorescence, because it is only partly exposed to solvent and because it is quenched by its proximity to a disulfide bridge. The Trp-19 residue that remains is therefore mainly responsible for fluorescence emission at λ_ex_ = 294 nm. It is located at the bottom of the calyx (Figure 1) and is sensitive to changes in the polarity in its vicinity, which are accompanied by a shift or quenching of the fluorescence emission [46,49]. Fluorescence spectroscopy can therefore be used as a method to measure changes to the micro-environment of the Trp-19 residue, which occur upon binding of ligands. It is the preferred method for studying the binding stoichiometry of the β-LG–ligand complexes [29,30].

The fluorescence emission maximum of β-LG is 332 nm, while the emission maximum of free tryptophan is 354 nm. The binding of a ligand that increases the polarity of the environment surrounding the Trp-19 residue causes a red shift of the emission maximum. On the contrary, a blue shift indicates that the tryptophan is exposed to a more apolar environment [29,50].

Binding with a ligand can also affect the fluorescence emission, changing its intensity. By measuring the variation in intensity against the ligand concentration, for example, in a titration experiment, the binding constant can then be calculated using a Stern–Volmer equation (Equation (1)) [49,51]. *F*_0_ is the unquenched fluorescence intensity, *F* is the fluorescence intensity measured upon the addition of a concentration of ligand [*L*] and *K_SV_* is the Stern–Volmer quenching constant.

(1) $$\frac{F_{0}-F}{F}=K_{SV}\times [L]$$

In the simplest case of static quenching of a single class of fluorophore, the Stern–Volmer plot is linear, whereas deviations from linearity indicate a combination of different quenching phenomena or fluorophores [18]. The binding stoichiometry may also be calculated from the Stern–Volmer plot [52].

While the principle behind the method is simple, other possible causes for the change in the fluorescence intensity should be accounted for, namely, the inner-filter effect, ground state complex formation and collisions [29]. In a 2011 review paper, De Weert and Stella outlined the importance of these phenomena [52]. Firstly, it must be demonstrated that the ligand or other compounds present in the sample do not have significant absorbance at the excitation or emission wavelengths. This is known as the inner-filter effect, and it occurs when fluorescence to or from the fluorophore is absorbed, reducing its intensity [29,52]. Studies are usually performed at low concentrations of both protein and ligand to avoid inner-filter effects [53]. Another simple approach is to use a solution of pure tryptophan as a control. Assuming that the ligand has no binding affinity towards free tryptophan, any fluorescence quenching in the control is attributed to the inner-filter effects or collisional quenching [49].

### 4.2. Fluorescent Probes

Extrinsic fluorescent molecular probes can also be used to analyze changes in protein morphology and ligand binding. The most commonly used molecular probe in protein–ligand binding studies is 8-Anilinonaphthalene-1-sulfonic acid (ANS). ANS binds to exposed hydrophobic regions on the protein surface and becomes fluorescent. This technique can be used to measure conformational changes in the protein structure that alter the surface hydrophobicity [13,54]. The addition of a ligand can displace ANS from the protein binding site, leading to a loss of signal. In this way, the displacement of ANS can be used to evaluate competitive binding of ligands [55,56].

### 4.3. Circular Dichroism Spectroscopy

Circular dichroism spectroscopy is a method used to measure changes to the secondary and tertiary structure of the protein. Circular dichroism (CD) is the difference in the absorbance of circularly polarized light in different directions. Random coil or denatured protein has only a small CD signal. On the other hand, well-defined structures, such as α-helix and β-sheets have characteristic CD spectra. Proteins rich in α-helix have a positive peak at 193 nm and negative bands at 208 nm and 222 nm, and proteins with antiparallel β-sheets have a broad negative band at 218 nm and a positive band at 195 nm. With the help of appropriate software, the CD spectrum of a protein can be deconvoluted to determine the percentage of each contributing structure [57,58].

When a ligand binds to a protein, it gains an induced circular dichroism, which can be measured. Additionally, the binding may induce changes to the protein structure, such as modifying the percentages of α-helix, β-sheet and random coil. These conformational changes may be analyzed by the changes to the CD spectrum [18,57].

When designing CD experiments, it is important to correct the effect of the solvent on protein conformation. Even a small amount of non-aqueous solvent added during titration experiments may have a significant effect. When using chiral ligands, it is also necessary to account for their own CD spectrum [57].

### 4.4. Isothermal Titration Calorimetry

Protein–ligand binding is in most cases accompanied by either the release or absorption of heat. Isothermal titration calorimetry measures the heat flux as a result of ligand binding. Typically, small aliquots of ligand solution are added by sequential injections (titration) at regular intervals, while the heat flux is measured. This technique provides a complete thermodynamic characterization, i.e., the binding constant as well as the stoichiometry, without the need for fluorophores or other additives [30,59]. A limitation of this technique is the low throughput, given the long time required to complete the full titration.

### 4.5. Crystallography

X-ray crystallography allows the study of the spatial arrangement of the ligand and the protein. In addition to confirming the binding, this method can provide accurate information about the binding sites and stoichiometry, but not the binding constants [33]. The application of this method can be limited by difficulties in obtaining diffraction-quality crystals. Crystals of biological macromolecules are generally small, very fragile and sensitive to degradation. For crystallization, very high purity protein is required. The nucleation phase of crystals of macromolecules requires high levels of supersaturation, which on the other hand is not ideal for the crystal growth phase [60,61]. β-lactoglobulin has been co-crystalized using the hanging-drop vapor diffusion method with different ligands, such as fatty acids [39,56,62] and retinoids [36].

The crystal structure of β-LG is also used in combination with other methods. In 2024, Ghosh et al. probed the binding of curcumin and folic acid to β-LG using 2D-protein NMR spectroscopy. The binding of ligands caused chemical shifts in specific amino acid residues. Mapping those perturbations on a crystal structure of β-LG allowed the authors to identify the binding site [48]. Stender et al. used the same method to assign binding sites for alginate oligosaccharides [63].

### 4.6. Turbidity

Many of the studied ligands have very poor aqueous solubility and tend to form aggregates, leading to turbidity. When protein–ligand binding is present, the turbidity has been shown to decrease. This is because some of the ligand concentration is bound to the protein in the form of a complex, reducing the amount of ligand that can aggregate. Several studies have used turbidity reduction upon the addition of protein as a simple indirect measure of ligand binding. Sample turbidity can be measured spectrophotometrically, generally at 500 nm. This method does not give information on the binding location or stoichiometry and is limited to hydrophobic ligands [37,56,64].

### 4.7. Molecular Docking and Molecular Dynamics Simulations

Molecular docking is an in silico method used to simulate the interaction between a ligand and a protein and to calculate the preferred binding site and orientation of ligands. This tool can also provide estimates for the binding affinity as well as the most important types of interactions and is often used in conjunction with non-computational methods [65]. The technique requires as an input a high-resolution representation of the 3D structure of the protein, which can either be obtained from online databanks, or otherwise must be determined experimentally. Search algorithms are then used to explore all possible conformations and orientations for the protein and the ligand to calculate the most energetically favorable conformations [66]. Molecular docking has been well established as a screening technique in drug discovery and is nowadays being used in a similar manner to identify the molecular targets of nutraceuticals [66]. In recent years, molecular docking has been used in combination with molecular dynamics simulations, to model the protein–ligand interaction under different conditions relevant to food processes, such as varying pressure and pH [67].

## 5. Types of Nanocarriers

**Figure 3:**
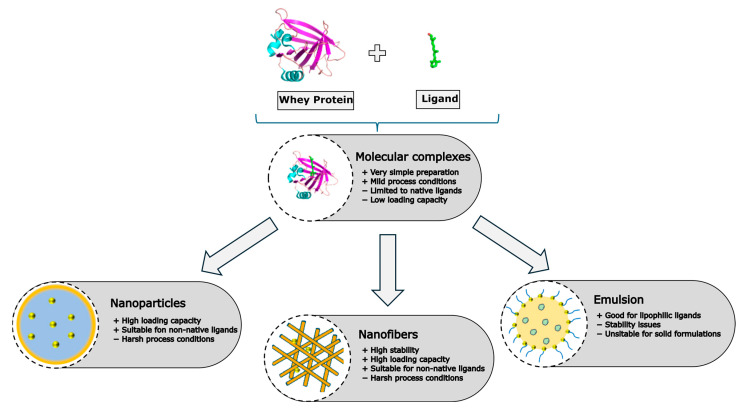
Advantages and limitations of different types of whey protein nanocarriers. Images adapted from [74].

Beyond simple molecular complexes, whey proteins can be formulated into different types of nanocarriers. Figure 3 shows the most important types of nanocarriers and lists some of the advantages and limitations.

Proteins, including whey proteins, can form supramolecular aggregates. These supramolecular assemblies are formed by the aggregation of protein molecules and can be exploited for the incorporation of ligands [68]. Aggregation can be induced by disrupting the native protein structure, for example, by thermal denaturation, via heat treatment [69]. Denaturation unfolds the protein, which exposes hydrophobic amino acid residues to the surface. The protein then aggregates due to electrostatic and hydrophobic interactions, forming nanostructures [10,55]. A limitation of the heat treatment method is that the ligand is usually exposed to heat, which causes degradation [70], although in some studies the ligand was added to pre-formed nanoparticles [71].

Besides temperature, the pH and the ionic strength also play an important role in protein aggregation behavior. Near the isoelectric point, the neutralization of surface charges tends to cause the ligand-bound protein to aggregate, which can be exploited to create nanoparticles [10]. Aggregates of whey protein can also be obtained by de-solvation, wherein an aqueous solution of the protein is added to an anti-solvent, inducing conformational changes and aggregation. The anti-solvent must then be removed or diluted. To avoid the breakup of the aggregates, a suitable crosslinker can be added [20]. Acidification and the addition of divalent cations, typically calcium, have been also used to induce aggregation [69].

Complexation with certain hydrophobic ligands, such as curcumin and α-tocopherol, also causes changes in the protein structure, inducing aggregation and nanoparticle formation without the need for heat treatment [44]. Epigallocatechin gallate (EGCG) has also been found to act as a crosslinker, binding multiple β-LG molecules [72,73]. This could be exploited to reduce the need for an added crosslinker [73].

### 5.1. Nanoparticles

Nanoparticles are nanostructures with particle sizes between 100 nm and 1000 nm. Owing to their subcellular size, nanoparticles have been exploited for encapsulation and targeted release. Protein nanoparticles consist of a dense polymeric matrix, allowing the entrapment of bioactives. Compared to simple molecular complexes, nanoparticles have better loading capacity due to having more hydrophobic patches. While molecular complexes are generally limited to the binding sites present in the native protein structure, nanoparticles offer the possibility to bind non-native ligands [20].

### 5.2. Nanofibrils

Like most proteins, β-LG self-assembles into amyloid-like nanofibrils under suitable conditions. These conditions usually involve prolonged heating at low pH (pH = 2) and low ionic strength, which causes the protein to unfold and hydrophobic residues that are buried in the interior of the protein structure in its native state are moved to the surface [75]. The protein is unfolded and hydrolyzed into polypeptides, which then aggregate unidirectionally into thin, fibrillar structures with a thickness of a few nanometers and a length in the micrometer range (1–10 µm) [76,77,78]. Low ionic strength and low pH promote the electrostatic repulsion between the protein molecules to avoid aggregation of native proteins [77]. Incubation in solvents that induce denaturation, such as urea or ethanol, may also cause fibrillation [78].

Similar to a crystallization process, fibrillation consists of a nucleation phase (or lag phase), a growth phase and a stationary phase [79,80,81]. The nucleation phase can be significantly shortened by the addition of pre-formed fibrils (“seeding”) [79].

The hydrolysis step of fibril formation can be tracked through SDS-PAGE analysis, by the gradual disappearance of the characteristic 18.3 kDa band of β-LG [54,82]. Fluorescent molecular probes can also be used to indirectly measure the formation of amyloid fibrils. Thioflavin T (ThT) is a fluorescent probe that selectively binds the molecular groove present on the surface of structures rich in β-sheet and its fluorescence is both enhanced and blue-shifted. At a constant ThT concentration, the fluorescence signal is linearly dependent on the fibril concentration, which allows the kinetics of fibril formation to be determined [83].

Protein amyloid-like nanofibrils share a “cross-β” structure, consisting of adjacent β-sheets, stacked perpendicular to the fibril axis. This structural motif has been associated with the high stability of protein nanofibrils [84]. Whey protein amyloid-like nanofibrils have increased surface hydrophobicity when compared with native whey proteins [82] and they are biocompatible and resistant to heat and to a wide range of pH values. These properties suggest that they can be used as encapsulants to protect sensitive biomolecules [76]. In addition, whey protein nanofibrils are excellent emulsifiers, which further hints at their potential as carriers of lipophilic bioactives [76,85].

It is possible to fine-tune the size and morphology of nanofibrils by manipulating the salt concentration, the pH and other process conditions. Shear forces and agitation increase the rate of self-assembly but result in shorter fibrils [75,76]. Low protein concentrations are required to obtain long straight fibrils. At higher protein concentrations, shorter fibrils are formed, with lower β-sheet content [80]. In 2023, Hoppenreijs et al. removed the disulfide bonds from β-LG through either chemical cleavage or recombinant substitution and showed that the intramolecular disulfide bonds stabilized the protein against fibrillation. On the other hand, the destabilization of the intermolecular disulfide bonds caused the formation of very small aggregates and hindered the formation of nanofibrils [86].

### 5.3. Emulsions

Due to the hydrophobic nature of many bioactive compounds, they can be stabilized in the oil phase of oil-in-water emulsions. The formation of a stable emulsion generally requires the addition of an emulsifier. Due to their amphiphilic nature, water binding, gelling and foaming capabilities, whey proteins are natural emulsifiers that can be applied to improve the stability in foods such mayonnaises and dressings [6,87,88]. Emulsions using whey proteins have also been shown to improve the stability of sensitive hydrophobic compounds, including vitamins [89] and curcumin [90], which suggests their application in beverages to stabilize sensitive additives.

### 5.4. Hybrid WPI Encapsulation Systems

The functionality of whey proteins as encapsulation systems can be further increased in combination with other types of encapsulants, most notably polysaccharides. For example, at acidic conditions near to its PI, β-LG may aggregate, which limits its application in many foods [87]. Whey proteins have been combined with polysaccharides to form binary systems, to improve the retention of nanostructure stability and bioactive retention during gastric digestion, allowing their release in the intestine. Protein–polysaccharide conjugates can be formed by Maillard reactions resulting in covalent bonds between protein amide groups and carbohydrate carbonyl groups [88]. Alternatively, binary hydrogels can be formed by taking advantage of interaction between both biopolymers [91]. Combining two or more encapsulation agents allows for greater flexibility and can produce nanovehicles with better properties than can be achieved with either encapsulation agent alone. On the other hand, using a combination of encapsulants increases the complexity of the system, leading to increased production costs.

Alginate has been added to whey proteins to form macroscopic beads [92], micro- and nanoparticles [93], and nanofibrils [94], to encapsulate both lipophilic and hydrophilic ligands. At low pH, hydrogel formation is favored by the opposite charges of alginate and whey proteins. To form hydrogels when the protein is negatively charged, Ca^2+^ ions are added [91,95]. The encapsulation efficiency is also improved by the alginate coating [95].

Whey proteins have also been combined with chitosan to form nanofibrils [96], nanocapsules [97] and emulsions [98,99] containing vitamins, polyphenols and other bioactive compounds. These hybrid scaffolds showed improved resistance during in vitro simulated gastric digestion. Protein–polysaccharide conjugates have been shown to possess better emulsifying capabilities than proteins alone [88].

## 6. Recent Advances in Ligand Stability in Whey Protein Nanostructures

One of the chief aims of encapsulation is to improve the stability of otherwise labile bioactive compounds. When the ligand is bound inside the protein scaffold, it is stabilized by the protein–ligand interactions formed, mainly hydrophobic interactions. It is also physically isolated inside the scaffold, protecting it from degradation reactions brought about by light, heat and other factors. Furthermore, the ligand is hindered from interacting with other ligand molecules, improving its solubility. This section reviews the recently published studies aimed at improving bioactive stability using whey proteins.

### 6.1. Polyphenols

Polyphenols play an important role in the color and taste of foods. Beyond their organoleptic properties, polyphenols also possess well-established health benefits. Polyphenols exhibit anticancer and antioxidant properties and help prevent cardiovascular and neurodegenerative disease [66]. Owing to their molecular structure, many phenolics have poor stability and solubility. As a result, antioxidant phenolics, such as curcumin and resveratrol, are a common class of encapsulation targets [18,19]. The interactions between proteins and polyphenols have been an object of great interest for the food industry. As an example, proteins from various sources, including milk caseins, have long been used in the beverage industry to precipitate polyphenols and improve the organoleptic properties of beer and wine [100].

Many different polyphenols have been shown to bind to β-LG. The affinity of polyphenols towards milk whey proteins generally increases with the molecular weight and number of hydroxyl groups [101]. The binding of polyphenols can have an important effect on the structure of the protein and consequently its properties. Some polyphenols have been shown to induce α-helix to β-structures transition. These changes have been associated with greater stability and lower gastric digestibility [102,103,104]. However, other studies have yielded conflicting results. Kanakis et al., reported that catechins caused an increase in the α-helix and β-structure contents and suggested that this may contribute towards the stabilization of the protein [101]. Yet other studies observed no significant changes induced by the binding of ligands such as curcumin [50] and resveratrol [50]. Understanding the effect of polyphenol binding on the structure of β-LG is crucial, as it has implications for its stability and lifetime in the gastrointestinal tract. Additionally, any changes in protein structure may affect the binding of further ligands [18].

Complexation with β-LG has been shown to improve the stability of different phenolic compounds. Table 1 shows a selection of published studies concerning the effect of complexation with β-LG on the stability of polyphenols. Sneharani et al. showed that complexation with β-LG in solution improved the half-life of curcumin, while encapsulation of curcumin in β-LG nanoparticles increased solubility [38]. The research group of Livney et al., studied the potential of β-LG nanoparticles for the protection of the polyphenol epigallocatechin gallate (EGCG) [70]. EGCG solution added to native β-LG undergoes slower oxidation than ECGC in buffer. When ECGC solution was added to β-LG pre-heated to 75 °C or 85 °C, the degradation rate of EGCG was much lower. On the other hand, when the protein solution was allowed to cool before the addition of the EGCG, the protective effect was similar to the native protein [70]. Taken together these results suggest that the protein unfolds upon heating, exposing hydrophobic binding patches to the surface, and refolds at least partially upon cooling. The authors further showed that β-LG-EGCG complexes could be freeze-dried and reconstituted. The β-LG encapsulated EGCG was colloidally stable and had improved sensorial properties when compared to non-encapsulated EGCG [105]. In a later study, the same group showed that complexation with β-LG confers greater stability and antioxidant activity during simulated digestion and in vivo [106]. Encapsulation in β-LG nanoparticles also retained the anti-proliferative activity of ECGC on Caco-2 cells [107].

A metric often used to evaluate the stability of polyphenols is the antioxidant activity, measured by radical scavenging assays [19]. Note that β-LG has significant intrinsic antioxidant capacity itself, so care must be taken to account for the contribution of the protein [108]. The effect of milk proteins on the antioxidant activity of polyphenols is not fully understood, but it has been suggested that the binding masks the hydroxyl groups and reduces their antioxidant activity when measured in vitro [104,109,110]. However, accounting for the improved lifetime of bound polyphenols, complexation may be a net positive in terms of storage stability and long-term antioxidant activity [104,111].

More recent studies have focused on the co-encapsulation of multiple different polyphenols, aiming to take advantage of the synergistic effects on their health benefits. In two 2022 studies, Zhang S. et al., evaluated the effect of β-LG on the stability of three antioxidant phenolics: ferulic acid, vanillic acid and quercetin [112]. They found that β-LG can simultaneously bind all three antioxidant phenolics and that the order of binding affects the binding affinity. These results suggest that the binding is non-competitive, with each ligand having a different binding site [112]. β-LG reduced the photodecomposition under visible light irradiation and heat decomposition at 25 °C and 35 °C [113]. Liu et al. reported that complexation with β-LG significantly increased the stability and bioavailability of different binary and ternary combinations of polyphenols: procyanidin B2 and dihydromyricetin [111]; EGCG, oxyresveratrol and piceatannol [109,114]; and chlorogenic acid and gallocatechin gallate [103]. The authors achieved even greater stability using heat-treated β-LG and encapsulation in nanoparticles [103,114].

Complexation with nanofibrils of whey protein has been shown to increase the antioxidant activity of curcumin [82]. The same studies also reported a major increase in the solubility of the poorly soluble curcumin.

**Table 1:** Published examples of whey-protein-based nanostructures used to encapsulate polyphenols, including the effect on the vitamin stability and the method used to determine stability.

Formulation	Encapsulation System	Effect of β-LG on Polyphenol Stability	Stability Assay	Ref.
Anthocyanins + β-LG + chitosan	Nanoparticles	− Improved stability during storage and simulated gastric digestion.	Antioxidant activity	[97]
Chlorogenic acid + gallocatechin gallate + β-LG	Molecular complexes + nanoparticles	− Improved stability in complexes, greatest in ternary complexes. − Nanoparticles have improved stability compared with complexes.	HPLC; antioxidant activity	[103]
Curcumin + β-LG	Molecular complexes + nanoparticles	− Improved stability and solubility.	UV-VIS	[38]
Curcumin + β-LG	Molecular complexes	− Improved antioxidant activity with β-LG, not necessarily due to binding.	Antioxidant activity	[108]
Curcumin + β-LG	Nanofibrils	− Improved solubility, storage stability and antioxidant activity.	UV-VIS; antioxidant activity	[82]
Curcumin + WPI + hyaluronic acid	Nanoparticles	− Improved stability and antioxidant activity.	UV-VIS spectrophotometry; antioxidant activity	[115]
Curcumin + WPI	Emulsions	− Improved light stability. Retention of ligand during simulated gastric digestion and release during simulated intestinal digestion.	UV-VIS spectrophotometry	[90]
EGCG + β-LG	Nanoparticles	− Slower oxidation.	UV-VIS spectrophotometry	[70]
EGCG+ β-LG	Nanoparticles	− Improved sensorial properties.	Sensorial panel	[105]
EGCG + β-LG	Nanoparticles	− Greater stability and antioxidant activity in simulated digestion and in vivo.	RP-HPLC; antioxidant activity	[106]
EGCG + OXY/PIC + β-LG	Molecular complexes	− Improved stability and solubility.	HPLC; antioxidant activity	[109]
ECG + PIC + β-LG	Molecular complexes + nanoparticles	− Protected the antioxidant activity. − Nanoparticles have improved stability compared with complexes.	HPLC; antioxidant activity	[114]
Ferulic acid + quercetin + vanillic acid + β-LG	Nanoparticles	− Improved stability.	UPLC; antioxidant activity	[112]
Ferulic acid + quercetin + vanillic acid + β-LG	Molecular complexes + nanoparticles	− Improved thermal- and photostability.	HPLC	[113]
Quercetin + β-LG + alginate	Molecular complexes + nanoparticles	− Improved stability during storage and simulated gastric digestion.	UV-VIS spectrophotometry	[93]
Resveratrol + β-LG	Molecular complexes	− Improved solubility.	UV-VIS spectrophotometry	[116]

### 6.2. Vitamins

Vitamins are essential micronutrients in diets. Vitamins are highly sensitive to degradation during processing and storage, and many are hydrophobic and suffer from poor water solubility. Vitamins therefore present interesting targets for encapsulation studies, aimed at improving their stability [117,118]. Table 2 shows a selection of such studies using complexation with whey proteins.

The protective effect of binding with milk whey proteins on retinoids and carotenoids has long been known [119]. It has also been shown to improve the stability against photo- and thermal degradation of vitamin E [37,120], vitamin B9 [17] and vitamin B12 [16]. The research group of Li Liang studied the protective effect of complexation with whey protein on several vitamins and phenolics. The same group later showed that β-LG can bind multiple ligands simultaneously [121]. In a later work, the authors achieved the simultaneous encapsulation of vitamin A, vitamin B9 and resveratrol in a β-LG–pectin complex and reported a greater protective effect on retinol than β-LG alone [122].

Simple complexation with β-LG has only achieved limited improvements in stability. More recent studies have focused on encapsulating vitamins in supramolecular nanostructures and on their release profile during digestion. Nanostructures should remain stable and retain their ligand during passage through the stomach and release their payload in the small intestine, where most vitamins are best absorbed. As discussed in Section 2, β-LG’s resistance to stomach proteases makes it an interesting delivery vehicle for vitamins. In 2013, Diarrassouba et al. demonstrated the use of β-LG nanoparticles to encapsulate riboflavin (vitamin B2) and vitamin D3. Vitamin D3 was encapsulated in a β-LG coagulum formed by acidification [123]. Encapsulated vitamin D3 showed increased storage stability and increased bioavailability in in vivo experiments [124]. Similar results were obtained by Abbasi et al. [125]. Lee et al. encapsulated vitamin D3 in WPI microgels obtained through cold-set gelation. In addition to increased thermal and storage stability, encapsulated vitamin D3 was retained during simulated gastric digestion and released during simulated intestinal digestion [126]. Madalena et al., used β-LG nanoparticles to encapsulate riboflavin (vitamin B2) and studied its release during simulated digestion [127]. The nanostructures remained stable during gastric digestion and degradation occurred in the simulated intestine. They were also shown to be stable in a simulated yoghurt environment during short-term storage [127]. Simões et al., developed a method to obtain nanoparticles of β-LG purified from commercial whey protein isolate, which was used to encapsulate riboflavin (vitamin B2) and quercetin as models of hydrophilic and hydrophobic compounds, respectively [128]. For both model compounds, nanoparticles had improved long-term stability [55] and were protected during simulated gastric digestion and their bioavailability was improved [129].

Other types of nanostructures have been evaluated for their potential as vitamin delivery vehicles, such as nanofibrils [130], liposomes [131] and emulsions [89]. Guo et al., studied the interaction between whey protein and multiple B-vitamins and showed that whey protein protected the vitamins both in emulsions and in spray-dried complexes [132].

**Table 2:** Published examples of whey-protein-based nanostructures used to encapsulate vitamins, including the effect on the vitamin stability and the method used to determine stability.

Formulation	Encapsulation System	Effect of β-LG on Vitamin Stability	Stability Assay	Ref.
Folic acid (vitamin B9) + β-LG/α-LA/BSA	Molecular complex	− Slower photodegradation.	Fluorescence spectroscopy	[41]
Folic acid (vitamin B9) + β-LG/α-LA/BSA	Molecular complex	− Slower photodegradation with all proteins. Most improved with β-LG.	Fluorescence spectroscopy	[17]
α-tocopherol (vitamin E) + β-LG	Molecular complex	− Slower photodegradation.	HPLC	[37]
Retinol + folic acid + resveratrol + β-LG	Molecular complex	− Slower degradation.	HPLC	[122]
Cobalamin (vitamin B12) + β-LG	Molecular complex	− Slower degradation under light, heat and in simulated gastric fluid.	HPLC	[16]
Riboflavin (vitamin B2) + β-LG	Nanoparticles	− Improved stability in simulated gastric digestion.	Fluorescence spectroscopy	[127]
Riboflavin (vitamin B2)/quercetin + β-LG	Nanoparticles	− Improved bioavailability and stability during long-term storage.	HPLC/UV-VIS spectrophotometry	[55]
Riboflavin (vitamin B2)/quercetin + β-LG	Nanoparticles	− Improved stability during storage and simulated gastric digestion.	Fluorescence spectroscopy HPLC	[129]
Vitamin D_3_ + β-LG	Nanoparticles	− Improved short-term storage stability.	HPLC	[125]
Vitamin D_3_ + WPI	Coagulum	− Improved solubility, long-term storage stability and bioavailability in vivo.	HPLC	[124]
Vitamin D_3_ + WPI	Nanoparticles	− Improved stability during storage and simulated gastric digestion.	UV-VIS spectrophotometry	[126]
α-tocopherol (vitamin E) + β-LG + alginate	Nanoparticles	− Improved retention during simulated gastric digestion.	UV-VIS spectrophotometry	[95]
α-tocopherol (vitamin E) + WPC	Nanoparticles	− Improved storage stability.	Fluorescence spectroscopy	[133]
α-tocopherol (vitamin E) + WPI	Nanoparticles + emulsions	− Improved long-term storage stability.	HPLC	[89]
Vitamin B_1_/B_2_/B_3_/B_6_ + WPC	Emulsion	− Improved stability in emulsions and spray-dried complexes.	Fluorescence spectroscopy	[132]
α-tocopherol (vitamin E) + WPI + chitosan	Emulsion	− Good stability during simulated gastric digestion and sustained release during simulated intestinal digestion.	UV-VIS spectrophotometry	[98]

## 7. Future Perspectives and Concluding Remarks

This review describes the structural features of β-LG behind the particular potential of whey protein as a vehicle for vitamins, polyphenols and other nutritional supplements. β-LG spontaneously binds otherwise labile bioactives, improving their stability and solubility. The conformational changes of β-LG offer the possibility of designing whey-protein-based nanovehicles for the targeted release of bioactives. Recent studies using simulated digestion have shown that β-LG is ideally suited for the protection of bioactives intended for intestinal release. Further, β-LG can self-assemble into a range of different types of nanostructures, which can be modulated to suit each application. These features, coupled with the wide availability and GRAS status of whey protein, have attracted a great deal of research interest. Beyond the food industry, β-LG has also attracted interest as a drug carrier, so that recombinant variants have been produced to bind novel ligands, in addition to the existing pool of known ligands.

An emerging trend can be identified from the cited literature: While there are many studies of the interaction between β-lactoglobulin and a variety of ligands, most are focused on determining the binding mechanism, the binding constants and stoichiometry and the effect of complexation on the protein structure. Relatively few studies are dedicated to the effect of whey protein encapsulation on the stability of the ligand itself. Often, ligand stability is shown to increase, especially in the short-term, but the ligand integrity is not demonstrated on the atomic scale nor for long storage periods. High-resolution 3D crystallographic structures of β-LG–ligand complexes can provide valuable insight into the exact mechanism of molecular encapsulation and confirm the extent to which it protects the ligand during food processing and storage. Additionally, while the protective effect of β-LG has been thoroughly demonstrated in the laboratory, there is a lack of studies on the formulation of the complexes as a functional solid product that can be scaled up. Future research should be aimed at obtaining stable protein–ligand complexes that can be formulated as a functional commercial food supplement.

The commercial application of whey-protein-based encapsulation may also face issues related to customer acceptance. Milk is a very prevalent food allergen and β-LG is known to be a major component involved in the allergic reaction. Additionally, there is a consumer trend away from animal proteins. Despite these issues, whey protein is a growing consumer trend, as a supplement and additive, with a global market exceeding USD 5 billion, with an estimated compound annual growth rate of 10.48% between 2022 and 2030 [134].

Although whey protein benefits from GRAS status, its use to encapsulate bioactive compounds, especially in nano-scale vehicles, is still subject to regulatory hurdles. The effect of nanoencapsulation on the absorption, biodistribution and toxicity of bioactives is still the subject of ongoing studies. Accordingly, the regulations for their safe commercialization are still being developed. The standardization of regulations across different markets also remains a challenge [9]. Despite these limitations, whey protein is a novel encapsulation agent, capable of providing atom-level protection of high-value labile ligands, which can hardly be achieved by traditional encapsulating agents.
